# Invaginating Structures in Synapses – Perspective

**DOI:** 10.3389/fnsyn.2021.685052

**Published:** 2021-05-24

**Authors:** Ronald S. Petralia, Pamela J. Yao, Dimitrios Kapogiannis, Ya-Xian Wang

**Affiliations:** ^1^Advanced Imaging Core, National Institute on Deafness and Other Communication Disorders/National Institutes of Health, Bethesda, MD, United States; ^2^Laboratory of Clinical Investigation, National Institute on Aging/National Institutes of Health, Bethesda, MD, United States

**Keywords:** spine, spinule, retina, *Drosophila*, planaria, invagination

## Abstract

Invaginating structures are common in the synapses of most animals. However, the details of these invaginating structures remain understudied in part because they are not well resolved in light microscopy and were often misidentified in early electron microscope (EM) studies. Utilizing experimental techniques along with the latest advances in microscopy, such as focused ion beam-scanning EM (FIB-SEM), evidence is gradually building to suggest that the synaptic invaginating structures contribute to synapse development, maintenance, and plasticity. These invaginating structures are most elaborate in synapses mediating rapid integration of signals, such as muscle contraction, mechanoreception, and vision. Here we argue that the synaptic invaginations should be considered in future studies seeking to understand their role in sensory integration and coordination, learning, and memory. We review the various types of invaginating structures in the synapses and discuss their potential functions. We also present several new examples of invaginating structures from a variety of animals including *Drosophila* and mice, mainly using FIB-SEM, with which we trace the form and arrangement of these structures.

## Introduction

Invaginating structures are small outward projections found in a diverse array of cell types (Bastiani and Goodman, 1984; Petralia et al., 2015; Wood et al., 2021), including synapses of neurons of almost all animals (reviewed in Petralia et al., 2015, 2016, 2017, 2018). The invaginating structures involve cell membranes of two different cells, with the outward projection – the invaginating structure – from one cell being surrounded by the invaginated membrane of the other cell. Therefore, in cross-sectional views of transmission electron microscopy (TEM), the invaginating structures can appear as double membrane-covered vesicles. In neuronal synapses, the invaginating structures can be divided into two main groups depending on the presence or absence of active zones.

Invaginating structures can be important in synapse physiology, yet they often have been overlooked in studies of synaptic function. This is especially true for the smaller spinule types of invaginating structures because they are difficult to identify without TEM, and even with standard 2D TEM, the origins of the invaginating structures are often obscure. Today, super-resolution and other specialized light microscopy techniques allow better visualization of these invaginating structures in synapses (Ueda and Hayashi, 2013; Zaccard et al., 2020). Moreover, the new wave of 3D EM methods such as focused ion beam-scanning electron microscopy (FIB-SEM) makes tracing of these invaginating structures possible. These approaches are inspiring scientists to examine the role of invaginating structures in synapses and neurons. In this perspective, we describe some of the more interesting examples of invaginating structures including several new examples from across the animal kingdom. We also discuss the latest ideas about how they may be central to the regulation of synaptic and neuronal function.

## Results and Discussion

### Invaginating Structures Associated With Mechanoreception and Photoreception (Figure 1A)

**FIGURE 1:**
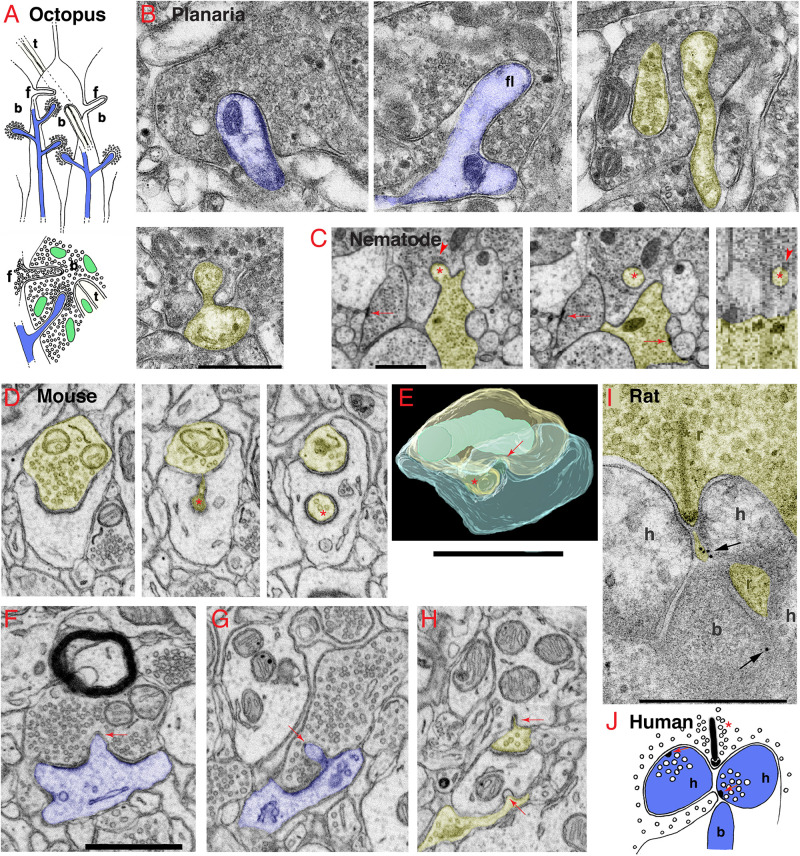
Invaginating structures are common in animal synapses. **(A)** Drawings recapitulating the octopus’s large *en passant* photoreceptor terminals, called “bags” (*b*) or “carrots.” The bags are filled with synaptic vesicles (shown in lower drawing) and contain three types of invaginating structures from three different sources, including: (1) postsynaptic spines (*blue*) with a dense layer of synaptic vesicles surrounding the deeply invaginating spine heads; (2) presynaptic terminals, also called “finger twigs” (*f*), which are filled with synaptic vesicles (lower drawing), invaginating from adjacent bags; and (3) “tunnel fibers” (*t*), which are one or more nerve trunks passing in a “tunnel” through the bag at ∼right angles to the invaginating spines and originating from small neurons called “microneurons.” Mitochondria are *green*. Drawings are from Petralia et al. (2017) with slight modifications. **(B)** Electron microscopy (EM) images of the planaria brain synapses. The invaginating structures include an invaginating postsynaptic dendrite (*blue*, left image), an invaginating filopodium (*f*, middle image), and interdigitating axon terminals (*yellow* and *uncolored*, right image). In the EM image on the left in the 2nd row, an unidentified projection invaginates into an axonal terminal (*yellow*) with large dense-cored vesicles. **(C)** EM images show an invaginating structure from the ventral ganglion of the nematode, *Pristionchus pacificus* (Bumbarger et al., 2013; serial cross-section online data set in Neurodata OCP). An invaginating structure (*asterisk*) originates from an axon terminal (*yellow*), which is one of two vesicle-filled terminals that form typical nematode dyadic synapses with a presynaptic density (*arrows*) centered between two postsynaptic processes (lacking PSD; White et al., 1986; Hall and Russell, 1991). The invaginating process enters into the base of a neurite extending from a neuron soma of the ventral ganglion (cell matches descriptions of neurons by position and structure; Ware et al., 1975; White et al., 1986). A possible junction may occur on the dorsal aspect of the invaginating process where the membranes appear denser and there are unidentified subsynaptic structures (*arrowheads*) in the postsynaptic cell. The left two images are transverse sections (z positions 2017 and 2019 in the image stack), and the right image is a digitally reconstructed parasagittal section. **(D–H)** Invaginating structures in the mouse nucleus accumbens. **(D)** An invaginating presynaptic terminal (*yellow*). The z positions in the FIB-SEM image stack are 144, 202, and 237 for the three images. The main part of the terminal partly invaginates into the cup-shaped postsynaptic process, and it then invaginates a portion of the terminal deep within the postsynaptic process (*asterisk*). **(E)** A 3D reconstruction of a similar invaginating presynaptic (yellow) terminal (asterisk) from the same data set in panel **(D)**, turned about 90 degrees relative to the structure in panel **(D)**. The postsynaptic membrane also invaginates a short spinule (*arrow*) into the presynaptic terminal (yellow), similar to the one shown in panel **(F)**. The 3D reconstruction is reprinted, after slight modification, from Delgado et al. (2019). **(F,G)** Examples of postsynaptic (*blue*) membrane invaginating short spinules (arrows) into presynaptic terminals. The EM image in panel **(F)** also includes a myelinated axon in which the glial cytoplasm (oligodendrocyte) partly invaginates into the axon. **(H)** Two presynaptic terminals invaginate short spinules (arrows) into dendrites (adjacent EM image in *z* position to this EM image is published in Delgado et al., 2019). **(I)** ImmunoEM of rat brain synapse. Immunogold localization (*arrows*) of GABA-A receptors in invaginating structures in the rod spherule of the rod photoreceptor synaptic terminal complex (*r*) in the rat retina. As is typical in vertebrate retinas, a complex of processes (*b*, *h*) from bipolar and horizontal cells invaginate into the terminal adjacent to the active zone identified by the presynaptic ribbon (*asterisk*). The immunogold labeling for GABA-A (*arrow*) is concentrated between a horizontal cell process and a small projection extending from the presynaptic rod cytoplasm and directly subjacent to the active zone. **(J)** Drawing shows that in the human retina, rod photoreceptor synaptic terminals have a ribbon (*asterisk*) synapse with an invaginating structure from one bipolar and two horizontal cells (*b*, *h*) plus a small projection of cytoplasm from the rod terminal. Horizontal cell processes can form synapses (*red arrows*) with the rod terminal and its projection and with the bipolar cell process; they contain large vesicles and presynaptic densities (Linberg and Fisher, 1988). Panels **(I,J)** are reprinted from Petralia et al. (2017) with slight modifications. Scale bars **(B**,**I)** = 500 nm, **(C,E,F)** (apply **D**,**G,H**) = 1 μm.

Some of the most elaborate arrangements of invaginating structures are found in synapses of the circuits involved in processing mechanoreception or photoreception and are adaptations to allow animals to respond very rapidly to changing environmental mechanical and visual stimuli (Petralia et al., 2017). They include various combinations of invaginating presynaptic terminals and postsynaptic spines (Figure 1A). The most amazing example is seen in cubozoan jellyfish, which have eyes as elaborate as those of higher animals even though they lack brains! These jellyfish exhibit complex behaviors involving vision, such as avoiding obstacles, prey capturing, and complex mating behaviors (e.g., Nilsson et al., 2005). They possess photoreceptor cells with prominent invaginating spines from postsynaptic cells or other photoreceptor cells (Gray et al., 2009). This suggests that the invaginating synapse was one of the earliest functional developments in animal nervous systems, even forming prior to the evolution of any form of “brain.” Invaginating postsynaptic spines can be found in some invertebrate sensory cell synapses such as in the octopus statocyst involved in balance and hearing, and mechanoreceptor cells involved in the defensive gill-withdrawal reflex of the sea hare, *Aplysia* (Bailey and Thompson, 1979; Bailey et al., 1979). Interestingly, the invaginating spines of *Aplysia* have twice as many presynaptic vesicles as non-invaginating ones; the authors attribute this to the high degree of synaptic plasticity related to the reflex (Bailey and Thompson, 1979; Bailey et al., 1979). Hair cell synapses of the tunicate, *Ciona intestinalis*, can have invaginating structures at their base and these can be postsynaptic, presynaptic, or both (reciprocal – with presynaptic vesicles on both sides of the synapse; Rigon et al., 2018). In the octopus (Figure 1A), the photoreceptor terminals form large bag- or carrot-shaped structures that are filled with presynaptic vesicles and contain (1) invaginating postsynaptic spines, (2) presynaptic vesicle-filled “finger twigs” from adjacent carrots, and (3) “tunnel fibers” from small neurons (Dilly et al., 1963; Case et al., 1972). Structures like “finger twigs” also are found in squid photoreceptor terminal “carrots.” Neither the finger twigs nor tunnel fibers show any distinctive signs of chemical synapses (no definitive active zones with densities), except for the synaptic vesicles in the finger twigs. Due to their deep invagination of the photoreceptor terminal, these structures are instead ideally arranged to mediate electrical field/ephaptic conduction (Cohen, 1973; Haghighat et al., 1984; Petralia et al., 2017).

### Simple Brains (Figures 1B,C)

Flatworms are the simplest animals with bilateral symmetry, a head, and a brain. Even at this earliest stage in brain evolution, a variety of invaginating structures are evident including at postsynaptic dendrites or other cellular processes with or without synaptic active zones, and various presynaptic terminals invaginating and interdigitating with other terminals (Figure 1B; Petralia et al., 2015). Nematodes have a simple nervous system with a minimal “brain” structure composed of a circumpharyngeal nerve ring and associated neuron clusters including the ventral ganglion (White et al., 1986). Recent studies show that nematodes have a variety of types of spine synapses similar to those found in vertebrates (Cuentas-Condori et al., 2019). White et al. (1986) showed several examples of presynaptic terminals invaginating into postsynaptic processes, and postsynaptic processes (spines) invaginating into presynaptic terminals, as well as a motoneuron terminal invaginating into an interneuronal cell body. In Figure 1C, a presynaptic terminal invaginates a structure into the base of a neurite extending from a neuronal soma in the ventral ganglion. A possible junction may occur on the invaginating structure where the membranes appear denser and there are unidentified subsynaptic structures in the postsynaptic cell.

### Vertebrate Brains (Figures 1D–H)

Invaginating structures are rather common in synapses of the vertebrate brain. For example, in a recent study of the human temporal cortex, Rollenhagen et al. (2020) found examples of postsynaptic spines invaginating into presynaptic terminals. They also found examples of presynaptic terminals with active zones and large non-synaptic structures from presynaptic terminals, both of which invaginate into dendrites. We have examined a FIB-SEM dataset from mouse nucleus accumbens showing various examples, including (1) postsynaptic spinules invaginating into presynaptic terminals, (2) invaginating structures from presynaptic terminals forming cup-shaped synapses with a more deeply invaginating portion, and (3) short presynaptic spinules invaginating into dendrites (Figures 1D–H). These will be discussed below in relation to the published literature.

Spinules from the postsynaptic spine invaginating into the presynaptic terminal (Figures 1E–G) have been described in many areas of the mammalian brain especially in the hippocampus (Westrum and Blackstad, 1962; Spacek and Harris, 2004; Yao et al., 2005; Tao-Cheng et al., 2009). An interesting example was documented in mouse barrel cortex, where some postsynaptic spines invaginate fully into the presynaptic terminals and then appear to extend a thick process, filled with various vesiculate structures and filaments, deeper within the terminal (Rodriguez-Moreno et al., 2018, 2020). Spinule formation is enhanced in hippocampal slice cultures following stimulation to induce long-term potentiation (LTP; Tao-Cheng et al., 2009) suggesting that spinules recycle extra postsynaptic membrane formed during enhanced synaptic activity. Indeed, some spinules are associated with the formation of the large, mushroom-shaped spines during synaptic plasticity such as that following LTP (Petralia et al., 2014, 2015, 2018). These mushroom-shaped spines enlarge since more membrane is added as additional glutamate receptor molecules are incorporated into the postsynaptic membrane; this increase in receptors likely enhances synaptic transmission. Apparently, this added membrane causes the PSD to become perforated in correlation with the increased density of glutamate receptors (Ganeshina et al.,2004a,b). At this point, a spinule may form at the perforation, invaginate into the presynaptic terminal (Figure 1F), and transfer excess postsynaptic membrane into the presynaptic terminal (Spacek and Harris, 2004; Tao-Cheng et al., 2009; Petralia et al., 2014, 2015, 2018). Coated pits often are seen at the ends of spinules (Westrum and Blackstad, 1962; Spacek and Harris, 2004; Yao et al., 2005; Tao-Cheng et al., 2009), mediating removal and absorption of spinule ends into the terminal. And recent studies with enhanced resolution 3D light microscopy have confirmed that neuronal activity induces spine-derived spinule elongation (Zaccard et al., 2020).

**FIGURE 2:**
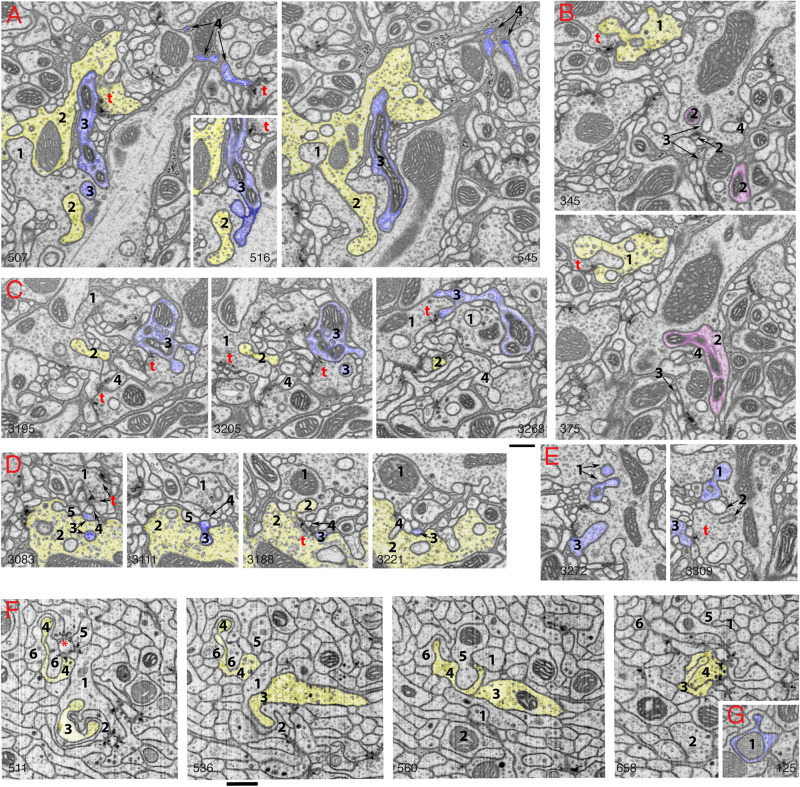
Invaginating structures in the *Drosophila* brain. Examples are FIB-SEM image stacks of the protocerebral bridge **(A–E)** and mushroom body **(F,G)**. *Blue*, dendrite; *yellow*, axon; *magenta*, either dendrite or axon or both. Axon terminals were defined by accumulation of synaptic vesicles or were traced to a presynaptic T-bar; dendrites were traced to a postsynaptic process. Invaginating structures are defined as outward projections. **(A)** Neurites 1 and 2 are large axon terminals that co-invaginate (neurite 1 invaginates into neurite 2 while neurite 2 invaginates into neurite 1). Neurite 3 is a dendrite that invaginates into axon 1, and neurite 3 is one of the two postsynaptic processes of a T-bar synapse (*t*) of axon 2. Neurite 4 is a dendrite that invaginates into a glial cell process; neurite 4 also is one of two postsynaptic processes at a T-bar synapse in an adjacent axon terminal (left image). **(B)** Neurite 1 is an axon terminal that invaginates into an adjacent axon terminal; neurite 1 is also postsynaptic at a T-bar synapse in the adjacent terminal (bottom image). Neurites 2, 3, and 4 invaginate into the same large axon terminal; 3 and 4 are small dendrites. Neurite 2 (*magenta*) was traced for a long distance (>4 μm). This neurite 2 displays features of both presynaptic and postsynaptic structures and forms at least two or three T-bar synapses as well as two or three postsynaptic processes with different synapses (not shown). **(C)** Axon terminal 1 is invaginated by axon terminal 2 and also invaginates another terminal. Neurite 3 is a dendrite that forms four spine-like structures, including one that forms a postsynaptic process at a synapse with terminal 1 and another that invaginates into a subjacent terminal. Neurite 4 is a dendrite that also invaginates into the same subjacent terminal. **(D)** A structure from axon terminal 2 invaginates into axon terminal 1, while structures from dendrites 3, 4 and 5 invaginate into terminal 2. **(E)** Neurites 1, 2, and 3 are projections from dendrites that invaginate into the same large axon terminal; neurite 1 has two invaginating structures. Neurite 3 also bears some T-bar like structures (not shown). Invaginating structures from axon terminals can be filled with synaptic vesicles as seen in panels **(A,B)**, or devoid of vesicles as evident in panel **(D)**. **(F)** Neurites 1-6 are all small axon terminals with relatively few synaptic vesicles. These axons invaginate with each other and also often cluster to form synapses on central dendrite processes. **(G)** An example of a dendrite (1) invaginating into an axon terminal. The number in the lower left or lower right corner of each micrograph indicates its z position in the FIB-SEM image stack. *t* = T-bar (only selected ones are labeled). Scale bars are 500 nm for panels **(A–E)** and **(F–G)**. Note that the protocerebral bridge neuropil **(A–E)** contains abundant invaginating processes from large axon terminals and dendrites, while the mushroom body neuropil **(F,G)** contains abundant invaginating processes from small axon terminals but few from dendrites.

Invaginating structures originating from presynaptic terminals in many animals vary from small spinules (Figure 1H) to larger structures and are often filled with presynaptic vesicles (Figures 1D,E). In the mammalian forebrain, some spinules that invaginate into presynaptic terminals originate from adjacent axons or presynaptic terminals, from ∼12% in the CA1 region of the rat hippocampus (Spacek and Harris, 2004) to ∼35% in the visual cortex of the ferret (Campbell et al., 2020). Invaginating structures from adjacent presynaptic terminals that are filled with synaptic vesicles often enter each other; these “pseudopodial indentations” or “PSIs” are described in some vertebrate synaptic terminals and can sometimes form complex intertwinings (Boyne and Mcleod, 1979; Boyne and Tarrant, 1982; see invertebrate examples in Figures 1B, 2). Such complex structures could act as “variable diffusion traps” to control levels of ions and other substances in the space between the processes (Boyne and Tarrant, 1982). Electrical stimulation of presynaptic terminals on the electrical organ of torpedo rays increases PSI frequency and size (∼27×; Boyne and Mcleod, 1979). Some inhibitory GABAergic terminals in the mammalian forebrain invaginate short structures into the postsynaptic cell. The postsynaptic membrane surrounding the invaginating structure contains an enzyme to synthesize cannabinoid that mediates a retrograde signal for tonic inhibition of synaptic activity (Yoshida et al., 2011; Omiya et al., 2015).

Cup-shaped spines are highly concave spines that wrap around partly or fully invaginating presynaptic terminals. They are common in cerebral cortex and hippocampus of mammals, and especially in the dentate gyrus (Desmond and Levy, 1983; Frotscher and Leranth, 1986; Petralia et al., 2017, 2018). Cup-shaped spines can be even more complex in the nucleus accumbens (Delgado et al., 2019; Yao et al., 2020), where the presynaptic terminal can continue in part as a deeper invagination with a synaptic active zone (Figures 1D,E; Delgado et al., 2019). Desmond and Levy (1983) found that high-frequency stimulation of entorhinal cortex input increases the number of concave spines in the dentate gyrus. Spines in CA1 slice cultures appear more cup-like after chemical induction of LTP (Nagerl et al., 2008), while the number of cup-shaped spines decreases after high-frequency electrical stimulation to induce LTP in CA1 slice cultures (Chang and Greenough, 1984). Cup-shaped spines appear to be more common in both slice and dissociated cultures compared to intact tissue (Roelandse et al., 2003; Mitchell et al., 2012; Petralia et al., 2017 and unpublished data). All of this suggests that formation of cup-shaped spines is a type of spine plasticity that is analogous in some ways to development of the large convex mushroom spines.

### *Drosophila:* Brain (Figure 2) and Neuromuscular Junctions

One of the most striking recent revelations about invaginating structures in synapses has occurred for the insect brain. When we first reviewed the invaginating structures of all animals in 2015 (Petralia et al., 2015), such structures were almost unknown for the insect brain.

The only examples were glia-derived capitate projections invaginating into photoreceptor terminals in the *Drosophila* eye (Prokop and Meinertzhagen, 2006) and some interaxonal invaginating structures (Petralia et al., 2015). Then, in 2018, utilizing FIB-SEM, (Gruber et al., 2018) described the synaptic spinules of the olfactory circuit of the *Drosophila* brain, and it became apparent that synaptic spinules are common. As can be seen for two areas of the *Drosophila* brain in Figure 2, there is a high abundance of invaginating neuronal processes into axonal terminals, derived from either dendrites or other axonal terminals. This pattern appears to be the rule for the *Drosophila* brain. Interestingly, some of the invaginating structures are derived from neurites with reciprocal synaptic functions, acting as both axon and dendrite. One such example is shown in Figure 2B: neurite 1 is a vesicle-filled axonal terminal but also forms one of the two postsynaptic elements of a photoreceptor terminal T-bar synapse, and neurite 2 was traced to different portions (not shown) containing postsynaptic processes or presynaptic T-bars. Similar reciprocal structures in interneurons are described for the ocellar photoreceptor terminal complex of *Drosophila* (Stark et al., 1989) that shows an example of a vesicle-filled interneuron invaginating into a photoreceptor terminal. However, photoreceptor terminals in both compound eyes and ocelli of *Drosophila* are invaginated mainly by specialized glial processes, rather than axonal or dendritic ones (reviewed in Prokop and Meinertzhagen, 2006; Petralia et al., 2015). Overall, the complexity of the invaginations in the *Drosophila* brain rivals or surpasses those found in the vertebrate brain, yet these neuronal invaginations in insect synapses were overlooked or missed by electron microscopists for the past 60 years!

Invaginations from presynaptic terminals also are common at neuromotor junctions including neuromuscular (NMJ) and secretomotor (such as glands) junctions (Petralia et al., 2017). These invaginating structures can either partially or fully invaginate into the postsynaptic cell. Such invaginating structures are part of mechanisms mediating rapid responses of skeletal muscle fibers. Because these invaginating structures also are found in NMJs of some slower muscles and glands, they might facilitate maintaining an enclosed space for exchange of regulatory factors. This function is best understood for NMJs of larval *Drosophila* skeletal muscle (reviewed in Deshpande and Rodal, 2016; Van Vactor and Sigrist, 2017, Guangming et al., 2020). A hundred-fold increase in muscle area occurs during larval growth (Deshpande and Rodal, 2016) and this must be accompanied by an equally impressive and matching growth in the NMJ; thus, this enclosed invagination area is a special arrangement to allow for the exchange back and forth across the synapse of a large number of different growth and regulatory factors to maintain this organization through development. For example, Wg (wingless; a Wnt ligand) is one of several regulatory proteins transported from the presynaptic terminal membrane via release of exosomes, probably from multivesicular bodies into the invagination intercellular space, that affect postsynaptic differentiation; other factors move retrogradely to affect presynaptic differentiation (Deshpande and Rodal, 2016). Another curious example is the transport of Arc1, important for synaptic plasticity, in capsid-like structures of Arc1 protein + mRNA within exosomes probably derived from presynaptic multivesicular bodies (Ashley et al., 2018).

### Invaginating Complexes of Processes (Figures 1I,J)

Some mechanoreceptor and photoreceptor cells in various invertebrates and vertebrates have large invaginations at their bases that contain a complex of both postsynaptic and presynaptic invaginating processes (Petralia et al., 2016, 2017). This is best known for the photoreceptor synapses of vertebrates (Figures 1I,J), in which the various processes are arranged within as well as subjacent to the invagination. Thus, they are in different positions and with different combinations of glutamate receptors within the area of glutamate spillover diffusion; GABA and ephaptic conduction are probably also involved here (Kramer and Davenport, 2015; Petralia et al., 2017). The main invaginating structures extend from bipolar and horizontal cells; their invagination and function are partly dependent on trans-synaptic complexes of proteins including calcium channel subunits and receptors (Kerschensteiner, 2017; Wang et al., 2017; Cao et al., 2020; Maddox et al., 2020; Tsukamoto et al., 2021). Invaginating horizontal cell processes form a type of reciprocal synapse including a feed-forward function along with negative feedback to provide lateral inhibition to help the brain modulate signals from groups of adjacent photoreceptor cells. The feedback mechanism from the horizontal cell processes to the photoreceptor cell may involve variable combinations of three different mechanisms: GABA (Figure 1I), proton (H^+^), and ephaptic transmission (electrical coupling between nerve processes not involving direct synapses) (Liu et al., 2013; Gardner et al., 2015; Kramer and Davenport, 2015; Petralia et al., 2017; Barnes et al., 2020; Hirano et al., 2020).

Horizontal cell processes vary in structure among vertebrates, and often have large vesicles of unknown function. Human horizontal cell processes at the rod photoreceptor terminal form definitive synapses (Figure 1J; Linberg and Fisher, 1988). Many fish have unusual spinules that invaginate into the photoreceptor cell from the horizontal cell processes, and they have enlarged ends with internal densities (Popova, 2014). These structures are numerous in the day but mostly gone at night. Popova (2014) suggests that they mediate feedback activity essential for the coding of antagonistic color information. They possibly have some role in postsynaptic neurotransmission and retract when glutamate receptors are activated (Weiler and Schultz, 1993

### Why Are Invaginating Structures So Important for Synapse Function?

We have discussed the various aspects of this question in greater detail in our previous reviews (Petralia et al., 2015, 2016, 2017, 2018). This is perhaps easier to answer for those invaginations with synaptic active zones containing presynaptic vesicles and postsynaptic densities. In these cases, the invagination creates a unique, isolated environment for biochemical exchange/activity between the presynaptic and postsynaptic structures. Depending on the structural arrangements, this can either improve the transmission of biochemical and/or electrical signals or sequester and isolate chemicals associated with plasticity between pre- and postsynaptic processes. One such example is the mossy terminal synapses of the hippocampus (Petralia et al., 2016, 2018). These large terminals are invaginated by large, modified compound spines called thorny excrescences, providing numerous active zones within the invagination (somewhat similar structures are found in the thalamus; Petralia et al., 2016; Pelzer et al., 2017). The cleft region is continuous and excludes glial processes. Overall, this specialized synapse is designed to have a higher net probability of release than typical cortical synapses (Henze et al., 2000). And as we have discussed, the invagination in the retinal photoreceptor synapses is highly organized with processes arranged at different distances and positions to take best advantage of neurotransmitter spillover and feedback mechanisms to affect the highly specialized visual responses. In some cases, an invaginating process without active zones is designed to modify neurotransmission, as we have discussed for presynaptic invaginating processes in inhibitory synapses in the mammalian forebrain and horizontal cell spinules in the fish retina. The *Drosophila* NMJ is the best studied example of a synaptic invagination providing an isolated and regulated local environment for chemical exchange to affect synaptic plasticity, as we discussed above. Finally, a large variety of small invaginating processes exists, and which are often broadly classified as “spinules,” lacking active zones and originating from postsynaptic, presynaptic, or glial components of the synapse. Many lines of evidence support various functions for these spinules in nutrient exchange, modulation/mediation of synaptic activity, and interneuronal signaling. Most intriguing and least studied are possible electrical field/ephaptic signaling effects (Faber and Pereda, 2018) that are likely facilitated by the invaginating structures (Gardner et al., 2015).

## Data Availability Statement

The raw data supporting the conclusions of this article will be made available by the authors, without undue reservation.

## Author Contributions

RP and PY organized the manuscript and figures. RP wrote the main text. RP, PY, and Y-XW provided the research data for figures. PY and DK edited the manuscript. All authors reviewed the manuscript.

## Conflict of Interest

The authors declare that the research was conducted in the absence of any commercial or financial relationships that could be construed as a potential conflict of interest.
