# Supplementary material for: Invaginating Structures in Synapses – Perspective
Source: Front Synaptic Neurosci. 2021 May 24;13:685052. doi: 10.3389/fnsyn.2021.685052 (PMC8180840; doi:10.3389/fnsyn.2021.685052)
Supplement: Supplementary file 1 [file Data_Sheet_1.docx]

**Supplementary Methods**

*Transmission electron microscopy (TEM) of rat retina and planarian brain*

Sections of adult rat retina and planarian brain were prepared as described previously (Petralia and Wenthold, 1999; Petralia et al., 2010; 2015, 2016, 2017; Yao et al., 2020). Briefly, rats were anesthetized and perfused with 4% paraformaldehyde and 0.5% glutaraldehyde; planaria were immersion fixed in the same fixative. Tissue was cryoprotected and frozen in a Leica EM-CPC and embedded in Lowicryl in a Leica AFS freeze-substitution instrument. Thin sections of the retina were immunogold labeled for GABA-A receptor, using Chemicon mouse antibody MAB341 and 10 nm gold (see Petralia et al., 2017 for further details). Thin sections of retina (Petralia et al., 2017) or planarian brain (Petralia et al., 2015, 2016; Yao et al., 2020) from two animals each were stained with uranyl acetate and lead citrate and examined in a JEOL JEM-2100 TEM. The image of the retina in figure 1 is reprinted from Petralia et al. (2017).

Petralia RS, Wenthold RJ (1999) Immunocytochemistry of NMDA receptors. Methods Mol Biol 128:73-92.

Petralia RS, Wang Y-X, Hua F, Yi Z, Zhou A, Ge L, Stephenson FA, Wenthold RJ (2010) Organization of NMDA receptors at extrasynaptic locations. Neuroscience 167:68-87.

*Nematode serial sections*

Data were obtained from a public online data set, using the Neuroglancer function of the Neurodata Open Connectome Project (<https://neurodata.io/ocp/>). Method details are described in Bumbarger et al. (2013). Specimens of the nematode, *Pristionchus pacificus* strain PS312 were high-pressure frozen in a Bal-tec HPM-10 and then freeze-substituted into epon in a Leica AM AFS2 freeze-substitution unit. Fifty nanometer sections were stained with uranyl acetate and lead citrate and imaged with a Gatan Ultrascan 4K camera mounted in a Tecnai T12 TEM. The data set contains 2762 transverse images with voxel size 3.9x3.9x50 nm. We examined the region of the ventral ganglion using the transverse sections as well as digitally reconstructed parasagittal sections. The information presented here is from s3://open-neurodata/bumbarger/bumbarger13/image, which is made available

under the ODC Attribution License (<https://opendatacommons.org/licenses/by/1-0/>).

Bumbarger DJ, Riebesell M, Rödelsperger C, Sommer RJ (2013) System-wide rewiring underlies behavioral differences in predatory and bacterial-feeding nematodes. Cell 152:109-119. We are grateful to Dr. Ralf J. Sommer for confirming the description of the methods.

*FIB-SEM data sets for mouse nucleus accumbens and Drosophila protocerebral bridge and mushroom body*

The FIB-SEM data set for nucleus accumbens was prepared in previous studies (Wu et al., 2017; Xu et al., 2017). The nucleus accumbens tissue was from an 11-month-old male mouse (C57/BL6J). Following intracardially perfusion with 2% glutaraldehyde and 2% paraformaldehyde, the brain was removed, post-fixed overnight, then cut into 50-μm sections. After additional steps of processing as detailed previously (Wu et al., 2017), the nucleus accumbens was dissected out, mounted, embedded in Epon, and trimmed. The samples were re-embedded in Durcupan, then imaged by a Zeiss Merlin FIB-SEM system (Xu et al., 2017). A focused gallium ion beam of 7-nA at 30 keV was used to remove 2-nm of the surface after each SEM image. The FIB-SEM data set for the nucleus accumbens used in the present study has 562 serial EM images with a voxel of 4x4x4 nm.

Wu Y, Whiteus C, Xu CS, Hayworth KJ, Weinberg RJ, Hess HF, De Camilli P. [Contacts between the endoplasmic reticulum and other membranes in neurons.](https://pubmed.ncbi.nlm.nih.gov/28559323/) Proc Natl Acad Sci U S A. 2017 Jun 13;114(24):E4859-E4867.

Xu CS, Hayworth KJ, Lu Z, Grob P, Hassan AM, García-Cerdán JG, Niyogi KK, Nogales E, Weinberg RJ, Hess HF. [Enhanced FIB-SEM systems for large-volume 3D imaging.](https://pubmed.ncbi.nlm.nih.gov/28500755/) Elife. 2017 May 13;6:e25916.

FIB-SEM data sets for *Drosophila* protocerebral bridge (3370 serial EM images) and mushroom body (685 serial EM images) were produced as described (Takemura et al., 2017; Xu et al., 2017; Xu et al., 2020). Brain tissue was from a 5-day-old adult female CantonS G1xw1118 *Drosophila*. Vibratome brain slices (200 mm) were fixed in 2.5% glutaraldehyde and 2.5% paraformaldehyde in 0.1 M cacodylate for 10-15 min before processing for freeze-substitution, followed by embedding in Durcupan resin. Smaller vertical posts were then trimmed to the region of interest – protocerebral bridge or mushroom body – guided by X-ray tomography data obtained by a Zeiss Versa XRM-510 and optical inspection under a microtome. A focused gallium ion beam of 7-nA at 30 keV was used to remove 2-nm of the surface after each SEM image. The acquired images formed a raw image volume, followed by post processing of image registration and alignment using a Scale Invariant Feature Transform (SIFT)-based algorithm. The aligned image stacks were further average binned to form a voxel of 4x4x4 nm. A masked Fourier filter was applied to remove streak artifacts.

Takemura S., Aso Y., Hige T. et al (2017) A connectome of a learning and memory center in the adult *Drosophila* brain. eLife 6:e26975.

Xu C.S., Hayworth K.J., Lu Z. et al (2017) Enhanced FIB-SEM systems for large-volume 3D imaging. eLife 6:e25916.

Xu C.S., Pang S., Hayworth K.J., Hess H.F. (2020) Transforming FIB-SEM systems for large-volume connectomics and cell biology. In: Wacker I., Hummel E., Burgold S., Schröder R. (eds) Volume Microscopy. Neuromethods, vol 155. Humana, New York, NY.

Figures 1A, E, I, J are reprinted with slight modifications, from previous publications. Figure 1E was published in Delgado et al. (2019), an open-access article. Figures 1A, I, J were published in Petralia et al. (2017); the latter publication was produced entirely from research performed in the U.S. Federal Government and as required, was published without assignment of copyright.
